# Epigenetic modulation of macrophage polarization prevents lumbar disc degeneration

**DOI:** 10.18632/aging.102909

**Published:** 2020-04-20

**Authors:** Yang Hou, Guodong Shi, Yongfei Guo, Jiangang Shi

**Affiliations:** 1 Department of Orthopaedic Surgery, Changzheng Hospital, Second Military Medical University, Shanghai 200003, China

**Keywords:** lumbar disc degeneration (LDD), aging, macrophage polarization, DNA methyltransferase 1 (DNMT1), transforming growth factor beta 1 (TGFβ1)

## Abstract

Inflammation plays an essential role in the development of lumbar disc degeneration (LDD), although the exact effects of macrophage subtypes on LDD remain unclear. Based on previous studies, we hypothesized that M2-polarization of local macrophages and simultaneous suppression of their production of fibrotic transforming growth factor beta 1 (TGFβ1) could inhibit progression of LDD. Thus, we applied an orthotopic injection of adeno-associated virus (AAV) carrying shRNA for DNA Methyltransferase 1 (DNMT1) and/or shRNA for TGFβ1 under a macrophage-specific CD68 promoter to specifically target local macrophages in a mouse model for LDD. We found that shDNMT1 significantly reduced levels of the pro-inflammatory cytokines TNFα, IL-1β and IL-6, significantly increased levels of the anti-inflammatory cytokines IL-4 and IL-10, significantly increased M2 macrophage polarization, significantly reduced cell apoptosis in the disc degeneration zone and significantly reduced LDD-associated pain. The anti-apoptotic and anti-pain effects were further strengthened by co-application of shTGFβ1. Together, these data suggest that M2 polarization of macrophages induced by both epigenetic modulation and suppressed production and release of TGFβ1 from polarized M2 macrophages, may have a demonstrable therapeutic effect on LDD.

## INTRODUCTION

lumbar disc degeneration (LDD) can cause debilitating low back pain, which restricts the activity level and quality of life of affected individuals [1–3]. Three major components—nucleus pulposus (NP), annulus fibrosus and cartilage end plates—comprise the lumbar disc, and apoptosis of NP cells secondary to excessive cartilage-specific extracellular matrix production has been found to be a central pathological feature of LDD [4]. This characteristic of LDD makes the prevention of NP cell death an attractive target for future therapies [5–9].

Macrophages, the main phagocytes in the body, figure prominently in the early stages of human growth and development. It was traditionally thought that macrophages are mainly phagocytotic in function, although later work has demonstrated numerous additional functions of macrophages [10–13]. Compared to the classical phagocytotic “M1” macrophages, the alternatively polarized macrophages, called “M2” macrophages, function as modulators of cellular and humoral immunity and as mediators of tissue repair and remodeling [10–13]. M2 macrophages highly express specific markers including CD206, CD163, arginase and CD301, and cytokines such as IL-4, IL-10 and IL-13. In contrast, M1 macrophages highly express CD86, nitric oxide synthase (iNOS) and reactive oxygen species (ROS), and their cytokine profile includes tumor necrosis factor alpha (TNFα), IL-1 β and IL-6 [10–13]. Transforming growth factor beta 1 (TGFβ1) is the most important growth factor enhancing tissue repair and fibrosis, and is believed to be produced and released by a subpopulation of M2 macrophages (M2c) in response to IL-10, in contrast to M2a macrophages which are primarily anti-inflammatory [14].

Prior work has shown that macrophages are the only inflammatory cells that infiltrate into the closed nucleus pulposus, and the number of macrophages is positively correlated with the severity of intervertebral disc degeneration [15]. Moreover, there is evidence to suggest that macrophages may either directly play a role in phagocytosis, or synergistically regulate lumbar disc metabolism through a neuro-immune mechanism. Likewise, macrophage dysfunction can cause the aggregation, chemotaxis and diffusion of inflammatory factors, leading to degradation of the extracellular matrix in the intervertebral disc, which in turn leads to lumbar disc degeneration [16–19]. However, whether macrophage polarization is critical for the development of LDD and by what mechanism it may affect LDD, remains to be experimentally tested. This question was addressed in the current study.

Here, we applied an orthotopic injection of adeno-associated virus (AAV) carrying shRNA for DNA Methyltransferase 1 (DNMT1) and/or shRNA for TGFβ1 under a macrophage-specific CD68 promoter to specifically target local macrophages in a mouse model for LDD. DNMT1 is an epigenetic modulator in macrophages, and has been shown to induce M2-priming of macrophages in vitro and in vivo [20]. We found that shDNMT1 significantly reduced levels of the pro-inflammatory cytokines TNFα, IL-1β and IL-6, significantly increased levels of the anti-inflammatory cytokines IL-4 and IL-10, and significantly increased the ratio of CD206+ M2 macrophages to CD86+ M1 macrophages. Likewise, shTGFβ1 did not significantly alter levels of these cytokines or the ratio of CD206+ M2 macrophages to CD86+ M1 macrophages, but did reduce TGFβ1 production and secretion. Application of shDNMT1 significantly increased lumbar proteoglycan and collagen II levels, regardless of co-application of shTGFβ1. ShDNMT1 significantly reduced cell apoptosis in the disc degeneration zone and reduced LDD-associated pain in mice, and these effects were significantly strengthened by co-application of shTGFβ1.

## RESULTS

### Preparation of AAVs that deplete DNMT1 and TGFβ1

**Figure 1:**
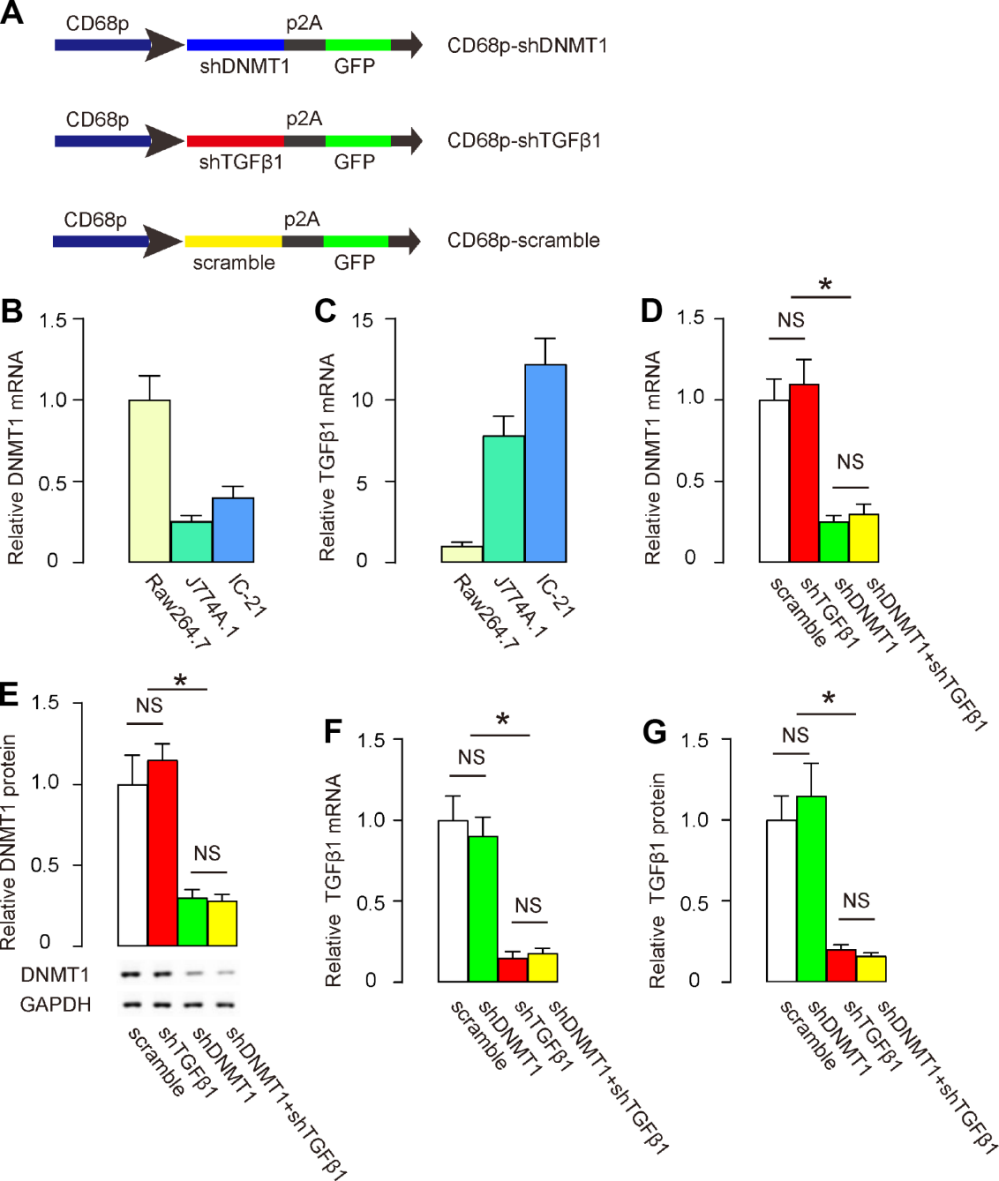
**Preparation of AAVs that deplete DNMT1 and TGFβ1.** (**A**) Schematic to show the structure of AAVs carrying shRNA for DNMT1 (shDNMT1) or shRNA for TGFβ1 (shTGFβ1) under a macrophage-specific CD68 promoter. The control AAV carried a scramble sequence under the CD68 promoter. The constructs were connected to a GFP reporter by p2A to allow co-expression by one promoter. (**B**, **C**) DNMT1 (**B**) and TGFβ1 (**C**) levels were examined in 3 mouse macrophage lines, Raw264.7, J774A.1 and IC-21, by RT-qPCR. (**D**–**G**) IC-21 was transduced with different AAVs. DNMT1 levels were determined by RT-qPCR (**D**) and by Western blot (**E**). TGFβ1 levels were determined by RT-qPCR (**F**) and by ELISA (**G**). *p<0.05. NS: non-significant. N=5.

In order to trigger M2 macrophage polarization and simultaneously suppress TGFβ1, we prepared two AAVs that carried either shRNA for DNMT1 (shDNMT1) or shRNA for TGFβ1 (shTGFβ1) under a macrophage-specific CD68 promoter. The control AAV carried a scramble sequence under the CD68 promoter. All constructs contained a GFP reporter (Figure 1A). Next, we examined DNMT1 and TGFβ1 levels in 3 mouse macrophage lines, Raw264.7, J774A.1 and IC-21. J774A.1 expressed the lowest DNMT1 (Figure 1B), while Raw2647 expressed the lowest TGFβ1 among the 3 cell lines (Figure 1C). Thus, IC-21 appeared to be the most appropriate line to test knockdown of DNMT1 and TGFβ1 by shRNAs. IC-21 was then transduced with either scramble-, shDNMT1-, shTGFβ1-, or combined shDNMT1 and shTGFβ1 AAVs. We found that shDNMT1 significantly decreased DNMT1 levels, with or without combined shTGFβ1, by RT-qPCR (Figure 1D) and by Western blot (Figure 1E). Transduction with shTGFβ1 did not alter DNMT1 levels by itself (Figure 1D, 1E). Moreover, shTGFβ1 significantly decreased TGFβ1 levels, with or without combined shDNMT1, by RT-qPCR (Figure 1F) and by ELISA (Figure 1G). Transduction with shDNMT1 did not alter TGFβ1 levels by itself (Figure 1F, 1G). These data confirmed the specificity of the prepared AAVs.

### CD68 promoter in AAVs allows specific targeting of macrophages

**Figure 2:**
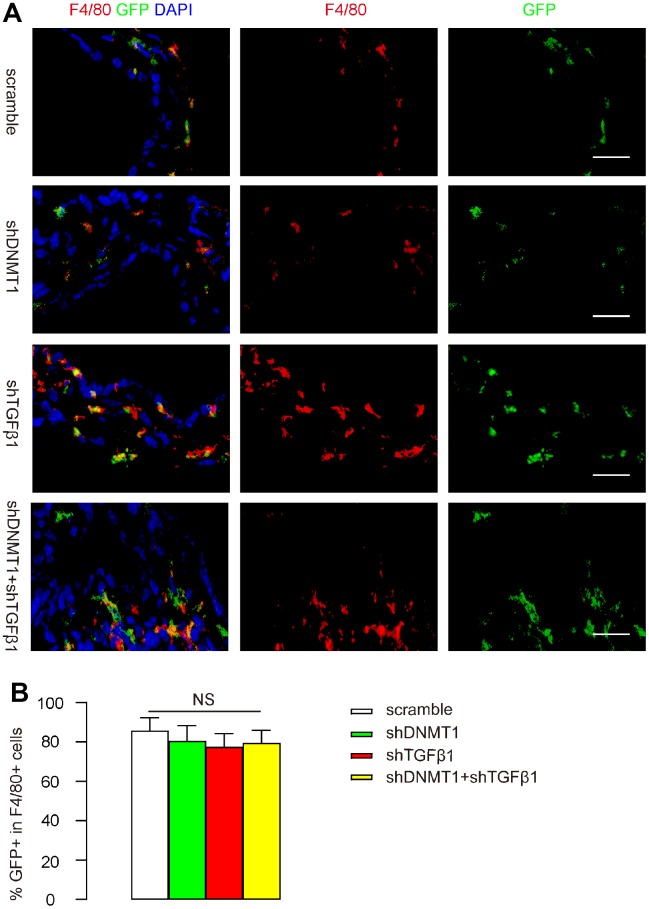
**CD68 promoter in AAVs allows specific targeting of macrophages.** After LDD was induced in mice, orthotopic injection of these AAVs into LDD-mice was performed. Mice in the scramble group received an orthotopic injection of 100 μl 10^11^ AAV-pCD68-scramble; mice in the shDNMT1 group received an orthotopic injection of 100 μl 10^11^ AAV-pCD68-shDNMT1; mice in the shTGFβ1 group received an orthotopic injection of 100 μl 10^11^ AAV-pCD68-shTGFβ1; mice in the shDNMT1+shTGFβ1 group received an orthotopic injection of 50 μl 0.5X10^11^ AAV-pCD68-shDNMT1 and 50 μl 0.5X10^11^ AAV-pCD68-shTGFβ1. Mice were kept for 4 weeks before analysis. (**A**, **B**) F4/80 staining and GFP signals are shown by representative images (**A**) and by quantification of the percentage of GFP+ cells in all F4/80+ cells (**B**). NS: non-significant. N=7. Scale bars are 20μm.

After LDD was induced in mice, orthotopic injection of these AAVs into LDD-mice was performed. Mice in the scramble group received an orthotopic injection of 100 μl 10^11^ AAV-pCD68-scramble; mice in the shDNMT1 group received an orthotopic injection of 100 μl 10^11^ AAV-pCD68-shDNMT1; mice in the shTGFβ1 group received an orthotopic injection of 100 μl 10^11^ AAV-pCD68-shTGFβ1; mice in the shDNMT1+shTGFβ1 group received an orthotopic injection of 50 μl 0.5X10^11^ AAV-pCD68-shDNMT1 and 50 μl 0.5X10^11^ AAV-pCD68-shTGFβ1. Mice were kept for 4 weeks before analysis. IHC showed exclusive detection of GFP in F4/80+ macrophages in all groups, and the percentage of F4/80+ macrophages that was positive for GFP (transduced cells) was quantified. These data are shown by representative images (Figure 2A), and by quantification (Figure 2B), and they suggest that the CD68 promoter in AAVs allows specific targeting of macrophages with high transduction efficiency.

### Effects of AAVs on cytokine production by macrophages

**Figure 3:**
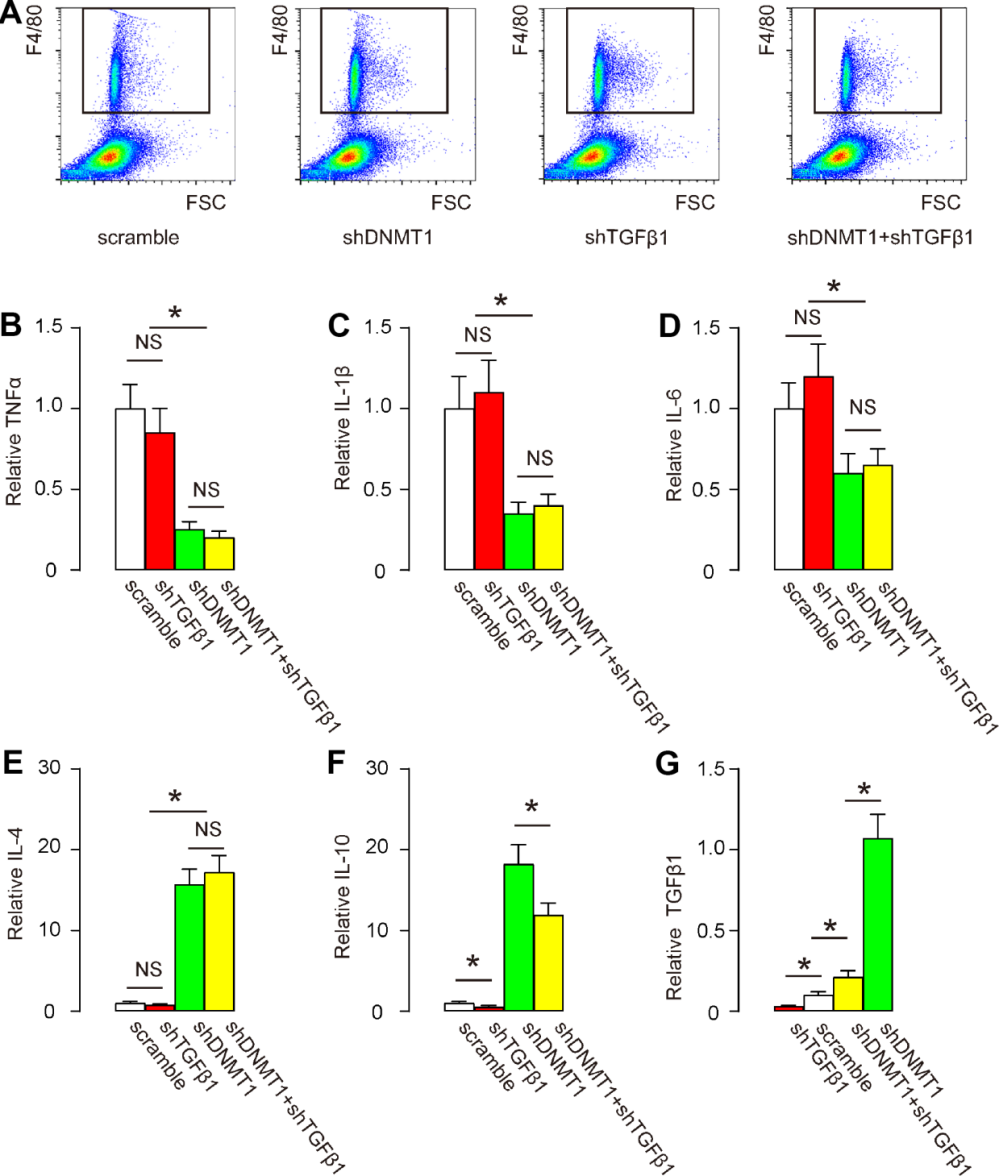
**Effects of AAVs on cytokine production by macrophages.** (**A**) F4/80+ macrophages in the degeneration zone were isolated by flow cytometry, as shown by representative flow charts. (**B**–**G**) ELISA for TNFα (**B**) IL-1β (**C**) IL-6 (**D**) IL-4 (**E**) IL-10 (**F**) and TGFβ1 (**G**). *p<0.05. NS: non-significant. N=7.

Next, we examined cytokine production by F4/80+ macrophages in the degeneration zone. Flow cytometry for F4/80 was performed as shown by representative flow charts (Figure 3A). We found that shDNMT1 significantly reduced pro-inflammatory cytokines TNFα (Figure 3B), IL-1β (Figure 3C) and IL-6 (Figure 3D), significantly increased anti-inflammatory cytokines IL-4 (Figure 3E) and IL-10 (Figure 3F), consistent with the known function of DNMT1 as an M2 macrophage polarizor. shDNMT1 also significantly increased TGFβ1 levels (Figure 3G), which may have resulted from its mediation of the M2 priming process, since TGFβ1 is mainly produced and released by M2c macrophages. shTGFβ1 alone did not significantly alter levels of most of these cytokines, including TNFα (Figure 3B), IL-1β (Figure 3C), IL-6 (Figure 3D) and cytokines IL-4 (Figure 3E), consistent with prior evidence that TGFβ1 is not a direct regulator of macrophage polarization. However, shTGFβ1 significantly decreased IL-10 (Figure 3F) and TGFβ1 (Figure 3G). Combination with shTGFβ1 did not attenuate the suppressive effects of shDNMT1 on pro-inflammatory cytokines (Figure 3B–3D) or the stimulatory effects on anti-inflammatory cytokines (Figure 3E); only the regulation by shDNMT1 on IL-10 and TGFβ1 was affected (Figure 3F, 3G), likely due to an interaction between IL-10 and TGFβ1.

### Effects of AAVs on macrophage polarization

**Figure 4:**
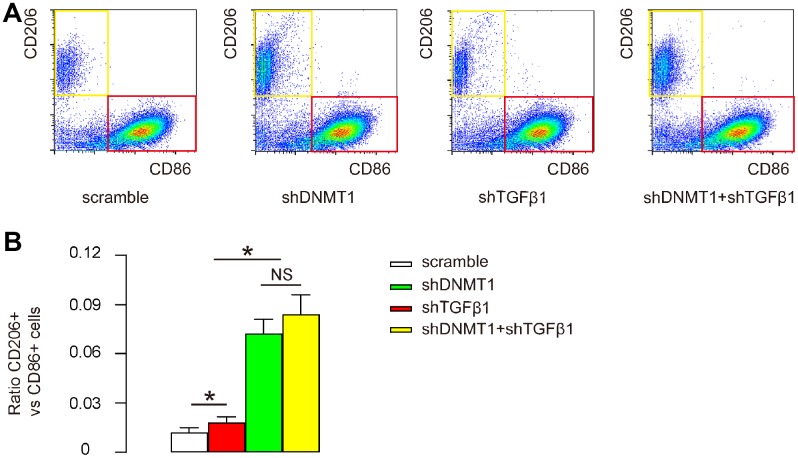
**Effects of AAVs on macrophage polarization.** (**A**, **B**) Flow cytometry for CD86 and CD206 on F4/80+ cells from the LDD zone, as shown by representative flow charts (**A**) and by quantification of the ratio of CD206+ M2 macrophages to CD86+ M1 macrophages (**B**). *p<0.05. NS: non-significant. N=7.

Macrophage polarization was examined by flow cytometry. CD86 is an M1 macrophage marker and CD206 is an M2 macrophage marker. Flow cytometry for CD86 and CD206 on F4/80+ cells was then performed as shown by representative flow charts (Figure 4A), and by quantification of the ratio of CD206+ M2 macrophages to CD86+ M1 macrophages (Figure 4B). Our data showed that shTGFβ1 only slightly increased this ratio, while shDNMT1, with or without shTGFβ1, significantly and comparably increased this ratio (Figure 4A, 4B). This finding suggests that shDNMT1 induced M2 macrophage polarization, which was not significantly affected by co-applied shTGFβ1.

### Co-application of shDNMT1 and shTGFβ1 reduces cell apoptosis in LDD tissue

**Figure 5:**
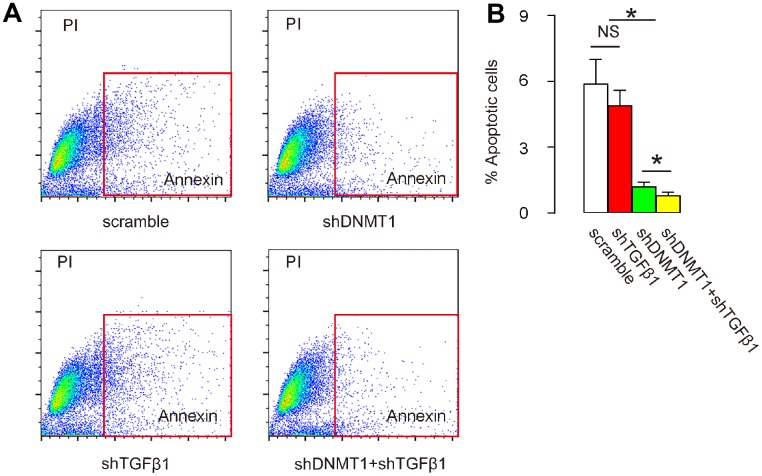
**Co-application of shDNMT1 and shTGFβ1 reduces cell apoptosis in LDD tissue.** (**A**, **B**) Cells isolated from mouse vertebral pulp and annulus fibrosus were dissociated into single cell populations for a flow-cytometry-based apoptosis assay, shown by representative flow charts (**A**) and by quantification (**B**). *p<0.05. NS: non-significant. N=7.

Next, we examined the effects of AAV treatment on the development and severity of LDD. Disc cell apoptosis is the central pathological feature of LDD, so cells from the mouse vertebral pulp and annulus fibrosus were dissociated into single cell populations for a flow-cytometry-based apoptosis assay. We found that the number of apoptotic cells in LDD was not significantly affected by shTGFβ1 alone, but was significantly reduced by either shDNMT1 alone or combined shDNMT1 and shTGFβ1, as shown by representative flow charts (Figure 5A), and by quantification (Figure 5B). Most importantly, the reduction in the number of apoptotic cells by shDNMT1 and shTGFβ1 appeared to be more pronounced than with shDNMT1 alone (Figure 5A, 5B). These data suggest that cell apoptosis in LDD tissue is most reduced by co-application of shDNMT1 and shTGFβ1.

### Effects of AAVs on spine proteoglycan and collagen II after LDD

**Figure 6:**
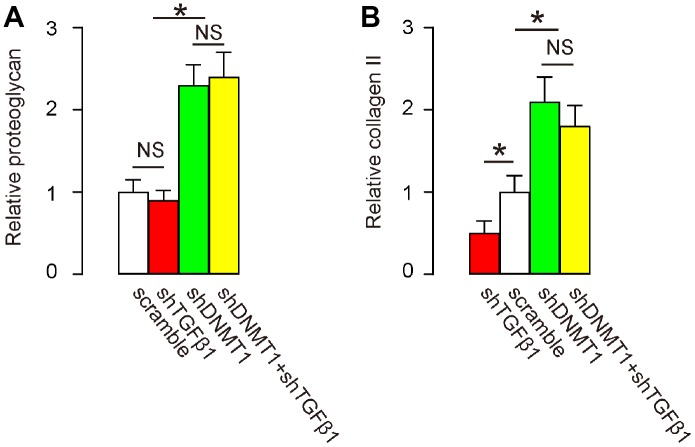
**Effects of AAVs on spine proteoglycan and collagen II after LDD.** (**A**, **B**) RT-qPCR for proteoglycan (**A**) and collagen II (**B**) in the LDD zone. *p<0.05. NS: non-significant. N=7.

Proteoglycan and collagen II levels in the spine are decreased in LDD proportional to disease progression and severity [21, 22]. We thus examined the levels of these factors by RT-qPCR after treatment. Our data show that levels of proteoglycan (Figure 6A) and collagen II (Figure 6B) were significantly increased by shDNMT1, regardless of whether shTGFβ1 was co-applied. These data suggest that combined shDNMT1 and shTGFβ1 may have improved therapeutic effects against LDD, compared to shDNMT1 alone.

### Effects of AAVs on LDD-associated pain

**Figure 7:**
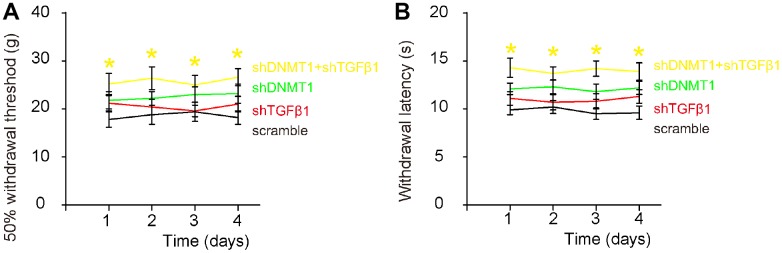
**Effects of AAVs on pain assessment after LDD.** Von Frey filament test was applied to evaluate the mechanical and thermal pain in 4 consecutive days. (**A**) Measurement of mechanically induced withdrawal threshold. (**B**) Measurement of thermally induced withdrawal latency of the paw. *(in yellow) p<0.05: shDNMT1+shTGFβ1 vs scramble. NS: non-significant. N=7.

Von Frey filament test was then applied to evaluate the mechanical and thermal pain in 4 consecutive days. Significant decrease in mechanically induced withdrawal threshold (Figure 7A) and significant in thermally induced withdrawal latency of the paw (Figure 7B) were only detected in mice that had received combined shDNMT1 and shTGFβ1, but not detected in mice that had received either shDNMT1 or shTGFβ1 alone, compared to mice that had received scramble. These data suggest that combined shDNMT1 and shTGFβ1 may have an effect against LDD-associated pain.

## DISCUSSION

LDD is highly associated with inflammation in the context of low back pain. Inflammation is not only associated with adverse symptoms related to the stimulation of nerve fibers that convey pain signals, but also has been implicated as the main factor responsible for LDD progression [16–19]. Macrophages are the most important immune players in diseased vertebral discs, and previous studies have provided immunohistochemistry evidence for the presence of macrophages in the LDD zone [16, 23]. Moreover, macrophages present at the disease site show phagocytic capability and contain lysosomes with collagen-degrading enzymes, which may be secreted by exocytosis leading to the breakdown of intercellular substances such as the disc matrix components, proteoglycans and collagens. Indeed, the continuous loss of proteoglycans and collagen is a hallmark of the pathogenesis of LDD, and is used as a marker for the severity of LDD [24].

The delicate phenotypic control of tissue macrophages is critical for proper tissue repair after injury and is very time-sensitive [25]. Too much M1 polarization results in severe inflammatory responses, severe tissue damage and poor recovery. However, too much M2 polarization may result in insufficient inflammatory responses and incomplete pathogen- and cell debris removal [25]. Importantly, M2-like polarization induces fibrosis, mainly mediated by TGFβ signaling [12]. Among the major cytokines, IL-10 has a distinct interaction with TGFβ. For example, TGFβ1 in combination with IL-4, promotes the differentiation of IL-10-producing T cells and macrophages [26]. On the other hand, IL-10 is the primary trigger of differentiation of M2c macrophages, which produce the majority of TGFβ1 [27–29]. Therefore, IL-10 levels in macrophages was compromised by shTGFβ1. Hence, in the current study, we co-blocked DNMT1 and TGFβ1 in macrophages. While DNMT1 suppression induced a general M2-like polarization in macrophages, specific inhibition of TGFβ1 may affect IL-10 production and secretion, which in turn reduces the generation of fibrotic M2c macrophages. Our results showed that co-blocking TGFβ1 did not attenuate the effects of DNMT1 inhibition on induction of M2-like macrophage polarization, but did reduce cell apoptosis and pain-associated MMP1, thereby promoting a favorable therapeutic outcome. Interestingly, TGFβ1 depletion resulted in decreased IL-10 production in macrophages, which may be due to the requirement for TGFβ1 to coordinate with IL-4 to activate IL-10 [26]. Co-suppression of TGFβ1 also attenuated the increase in IL-10 by DNMT1 suppression, perhaps for the same reason as above. Of note, IL-10 and its receptor are expressed by nearly all inflammatory cells, and can be either anti-inflammatory at lower levels or pro-inflammatory at higher levels [30]. Thus, attenuation of IL-10 signaling may help to put inflammation under control, which benefits the diseased tissue [30].

Decreases in both proteoglycan and collagen II that occur in disc matrix molecules are known to contribute to the loss of disc function with aging as well as disc degeneration [31, 32]. Surprisingly, the suppression of TGFβ1 did not appear to affect the salvage effects of inhibition of DNMT1 on proteoglycan and collagen II, which may in fact reflect compensatory effects of factors other than TGFβ1.

To the best of our knowledge, this strategy using orthotopic AAV-based gene therapy targeting both DNMT1 and TGFβ1 pathways is the first effective gene therapy for LDD. Further characterization of this method may result in clinical application in the future.

## MATERIALS AND METHODS

### Protocol approval and animal treatments

The present study was approved by the Research and Animal Ethics Association of Second Military Medical University. Specific pathogen free (SPF) Balb/c mice (aged 10 weeks, weight 20g) were supplied by the Animal Laboratory of the Academy of Medical Sciences (Beijing, China). Mice were randomly divided into 4 groups of 7 each. All groups received surgical induction of LDD, in which the sacrospinal muscles, spinous processes, supraspinous ligaments, interspinous ligaments and posterolateral halves of the bilateral zygapophysial joints of the lumbar spine were removed after intraperitoneal administration of ketamine 90 mg/kg and xylazine 10 mg/kg. Subsequently, mice in the scramble group received an orthotopic injection of 100 μl 10^11^ AAV-pCD68-scramble; mice in the shDNMT1 group received an orthotopic injection of 100 μl 10^11^ AAV-pCD68-shDNMT1; mice in the shTGFβ1 group received an orthotopic injection of 100 μl 10^11^ AAV-pCD68-shTGFβ1; mice in the shDNMT1+shTGFβ1 group received an orthotopic injection of 50 μl 0.5X10^11^ AAV-pCD68-shDNMT1 and 50 μl 0.5X10^11^ AAV-pCD68-shTGFβ1. Mice were kept for 4 weeks before analysis. A von Frey filament test was used to measure pain in mice. Briefly, the mice were put in a test box fitted with a wire mesh backing. Von Frey microfilaments were applied through the grid floor to the ventral surface of the hind paw of the injured hind limb of the mice. The filament was pressed till bent and wait for the mice to withdraw the hind leg without moving. During each test, the filament series was presented after the incremental procedure and the 50% response threshold for each mouse was calculated [33].

### Cell culture and AAV preparation

Several mouse macrophage lines (RAW264.7, J774A.1 and IC-21) were purchased from American Type Culture Collection (ATCC, Rockville, MD, USA) and were cultured in Dulbecco’s modified Eagle medium (DMEM, Gibco; Life Technologies, Carlsbad, CA, USA) with 10% fetal bovine serum (FBS, Sigma-Aldrich, St Louis, MO, USA), penicillin (100μg/ml) and streptomycin (250ng/ml) at 37°C, in a 5% CO_2_ atmosphere. AAV serotype 2 vectors were generated by transfection of human embryonic kidney 293 cells. shDNMT1 (5'-CCGGCCCGAGTATGCGCCCATATTTCTCGAGAAATATGGGCGCATACTCGGGTTTTTG-3'; target sequence: CCCGAGTATGCGCCCATATTT) and shTGFβ1 (5'-CCGGACTGCGGATCTCTGTGTCATTCTCGAGAATGACACAGAGATCCGCAGTTTTTT-3’; target sequence: ACTGCGGATCTCTGTGTCATT) used NIH published sequences. The scramble sequence was 5’-GGTATCTACTAGATGTACT-3’. Human CD68 promoter was obtained from Addgene (#34837, Addgene, Watertown, MA, USA) [34]. Transfection was performed with Lipofectamine 3000 reagent (Invitrogen, CA, Carlsbad, USA), according to the instructions of the manufacturer. The prepared virus was stored at -80°C. Titration of viral vectors was determined using a dot-blot assay.

### Apoptosis assay and flow cytometry

Cells were labeled with annexin V-FITC and propidium iodide (PI), using an apoptosis detecting kit (KeyGEN Biotech, Nanjing, China), and were analyzed by flow cytometry using FlowJo software (Flowjo LLC, Ashland, OR, USA). Flow cytometry-based macrophage sorting was conducted using PE-cy5-conjugated F4/80, APC-conjugated CD206 and pacific blue-conjugated CD86 antibodies (Becton-Dickinson Biosciences, Shanghai, China). Flow cytometry data were analyzed and presented with FlowJo software (Flowjo LLC, Ashland, OR, USA).

### Sampling and analysis by immunohistochemistry, western blot and ELISA

The lumbar spines of the sacrificed mice were dissected out. The vertebral pulp and annulus fibrosus were isolated for immunohistochemical, Western blot and ELISA analysis. For immunohistochemistry, the metaphyses of the vertebral pulp and annulus fibrosus specimens were fixed in 4% paraformaldehyde for 2 hours, then paraffin-embedded followed by being cut into 5-μm-thick sections. Immunohistochemistry was performed using fluorescence. GFP was detected by direct fluorescence. The primary antibody for immunohistochemistry was rat anti-F4/80 (Invitrogen, Cambridge, MA, USA; dilution: 1:300). Western blot was conducted in the standard fashion. The primary antibody for Western blot was rabbit anti-DNMT1 (Abcam, Cambridge, MA, USA; dilution: 1:1000). Secondary antibodies for immunohistochemistry and Western blot were cy3-conjugated anti-rat and HRP-conjugated anti-rabbit (Jackson ImmunoResearch Labs, West Grove, PA, USA), respectively. ELISA was performed using kits for TNFα, IL-1β, IL-6, IL-4, IL-10 and TGFβ1 (R&D System, Los Angeles, CA, USA).

### RNA isolation, quantitative polymerase chain reaction (RT-qPCR)

RNA extraction and cDNA synthesis were performed in the standard fashion. RT-qPCR primers were all purchased from Qiagen. Values were normalized against GAPDH, which proved to be stable across samples, and were then compared to experimental controls.

### Statistical analysis

All statistical analyses were carried out using GraphPad prism version 8.0 (GraphPad Software, Inc. La Jolla, CA, USA). Data were investigated using one-way ANOVA with a Bonferroni correction, followed by Fisher's exact test to compare 2 sub-groups. All values are shown as mean ± standard deviation (SD) and are considered significant if p < 0.05, not significant (NS) if p>0.05.
